# Exploring barriers and challenges in protecting residential fire-related injuries: a qualitative study

**DOI:** 10.5249/jivr.v11i1.1059

**Published:** 2019-01

**Authors:** Mohammadreza Shokouhi, Khadijeh Nasiriani, Hamidreza Khankeh, Hosein Fallahzadeh, Davoud Khorasani-Zavareh

**Affiliations:** ^*a*^Department of Emergencies and Disasters Health, Faculty of Health, Shahid Sadoughi University of Medical Sciences, Yazd, Iran.; ^*b*^School of Nursing & Midwifery, Shahid Sadoughi University of Medical Sciences, Yazd, Iran.; ^*c*^Department of Clinical Sciences and Education, Karolinska Institutet, Stockholm, Sweden.; ^*d*^Department of Biostatics and Epidemiology, Faculty of Health, Shahid Sadoughi University of Medical Sciences, Yazd, Iran.; ^*e*^Safety Promotion and injury Prevention Research Center, School of Public Health and Safety, Shahid Beheshti University of Medical Sciences, Tehran, Iran.

**Keywords:** Fires, Residential, buildings, Injury and death, Safety

## Abstract

**Background:**

Building fires can be a great threat to the safety of residents, and can lead to economic and social damage. Exploring the views of stakeholders is a great source for understanding the factors that affect fires. The purpose of this study was to explore stakeholders’ experiences of unintentional fire-related injuries in residential buildings in Iran.

**Methods:**

This qualitative study was carried out using grounded theory. The study was conducted in Iran, in 2017. The study participants consisted of 25 people including stakeholders who had practical experience/or were knowledgeable in the field of preventing and fighting building fires. Purposeful and theoretical sampling were used for data collection. Data were analyzed based on constant comparative analysis and according to recommendations by Strauss and Corbin.

**Results:**

"Lack of a comprehensive approach to prevention of fire-related injury" emerged as a core variable which impacted on residents' safety against fires. The findings were classified into four groups of challenges related to the structure of building, socio-economic challenge, residents of the building and rescue services.

**Conclusions:**

Based on participants` experiences, unintentional fire-related injuries in residential buildings are affected by cultural context and economic, social and geographical factors. Improving the safety against unintentional fire-related injuries in residential buildings requires multidisciplinary operations including both change and improvement of the building construction and change in the beliefs and practices of residents to increase safety against fires.

## Introduction

Accidental fires in residential building are risky events which have both direct and indirect effects on human life that affect the general community health.^1,2^ Fires are considered to be one of the major public health concerns all over the world, which yearly cause 200,000 deaths. Although fire-related injuries and deaths in low- and middle-income countries (LMICs) are most common, this issue is also of concern in high-income countries.^1^ Financial costs from fires over the world are yearly up to ten billion dollars which is estimated to be about one percent of Gross National Product in the world.^3^

People spend about half of their day in their homes. Houses are an important interface between lifestyle and health issues, and many injuries take place in this environment. The home is the second most common location for accidental injuries resulting in death.^4^ Fire in residential areas is one of the most important causes of deaths and injuries among all types of fires. For example, the cause of 75% of all fire-related deaths in the United States are fires in residential areas.^5^ Nearly three-quarters of the fatalities and injuries associated with fires are related to residential areas, resulting in economic damage. Although the number of deaths from fires has declined over the last few years, they are still considered as a major public health problem.^5^

Iran is located in a region exposed to a range of natural and man-made hazards.^6^ According to the surveys conducted in Iran in the 1990s, there were between 600 and 900 fires per million inhabitants per year in cities.^7^ A national study in Iran shows that, of 307,000 domestic injuries in 2000 to 2003, about 125,000 (41%) were related to fires and burns.^8^

In order to prevent morbidity, mortality and financial losses due to fires in residential areas, the causes of these fires should be identified.^9^ Human error and risky behaviors are the main causes of most fire events.^10,11^ On the other hand, in order to find the most effective interventions in prevention of unintentional residential fires, it is necessary to know about the people affected by the fires. ^12^ At present, our knowledge of how the residents behave in the event of a fire, how their experiences are shaped, and which factors determine the spread of fires based on their experiences is still limited in scope. There are a few studies in Iran that have mainly quantitative approaches and are inadequate in term of numbers. These studies cannot identify the experiences of the phenomenon of fires, related factors, human behaviors and fire-related injuries.

However, in terms of promoting fire safety policy, it is important to know why a particular incident causes a lot of casualties or why other catastrophic events have fewer losses.^13^ Several studies have shown that the use of stakeholders’ experiences is one of the most important ways of promoting the phenomenon of interest, as emphasized by the World Health Organization.^14-16^ Exploring the experiences of stakeholders is an important and valuable resource in extracting barriers and ways to promote a phenomenon. Accordingly, the purpose of this study was to explore stakeholders’ experiences of unintentional fire-related injuries in residential buildings in Iran.

## Methods 

This study was conducted using the grounded theory method, which is a qualitative method. This method is suitable when researchers are keen to discover new knowledge or to review a phenomenon with a fresh perspective.^17^ In each interview, all conversations were recorded and transcribed verbatim. It should be noted that the data were collected and analyzed simultaneously employing constant comparative analysis based on Strauss and Corbin principle.^17^

***Study setting***

The study was conducted at the national level in Iran, which is located in southwestern Asia and the Middle East. Iran is 1,648,195 square kilometers, and according to the 2016 census, has a population of 79,926,270.^18^ It should be noted that the setting of the study and where the experiences of the participants in this study shaped were residential buildings, firefighting centers, hospitals and offices.

***Participant selection and data collection***

The study lasted from December 2016 until December 2017. Participants included firefighters, physician and nurses involved in the provision of health care, the fire victims and those who experienced fires, and other stakeholders who had practical experience or theoretical knowledge about management and action after the fires that in total were 25 participants. This selection was based on purposive and theoretical sampling. The criteria for selecting participants were having fire-related experience and/or knowledge, and the desire and ability to share experiences. Selection of participants and data collection continued according to the principle of data saturation.

The interviews were conducted using semi-structured interviews. The main points of questions that only served as an interview guide were: "Please describe your experience of fires in residential buildings? What happened on the day of the fires? What did you do to prevent fire-related injuries? How do you keep safe from fire-caused injury? What are the barriers to fire safety in buildings? And how can you overcome these barriers? "How can the safety of residents be improved against fires? Then they continued with exploratory or in-depth questions such as "Explain more?" Take a sample from your own experience. The interview continued to saturate the concepts. The interview lasted from 40 to 60 minutes. This period was considered based on tolerance criteria, amount of information and willingness and agreement of the participants.

***Data analysis***

The basis of analysis in grounded theory method is constant comparative analysis, in which codes, category, and subcategories are compared for similarity and difference. In other words, the collection and analysis of data are done simultaneously and it is necessary for the researcher to become submerged in the data. This was done by listening, repeatedly, to the interviews and the descriptions given by the participants and with repeated data retrieval. The Strauss and Corbin (1998) method was used to analyze the data, which consists of three basic stages of open coding, axial coding and selective coding.^19^ In open coding, data are broken into their own components and compared in terms of similarities and differences.

It is necessary at this stage to write down the interview text and review it frequently until a general understanding of the concepts is reached. Then, the researcher identifies the main concepts contained in each line or paragraph and encodes each sentence.^17^ At this stage, data become abstract in the axial coding and the main classification of the data was performed. Constant comparison of the data was done, and the codes and the initial classes derived from the previous step were compared and the similar codes were conceptually merged.^17^ In the selective encoding, the categories, created in the previous step, are integrated to form the main variable. The purpose was to find the main category and establish relationships between the categories to determine the theoretical framework. At this stage, the core variable or the underlying process in the data, the manner and stages of occurring, its consequences, and its implications were revealed.^20^ In this study, the analysis of data was done according to the pattern proposed by Strauss and Corbin in 2008.

**Ethical consideration**

This study was approved by the Ethics Committee of Shahid Sadoughi University of Medical Sciences, Yazd on 20 December 2016.^21^ The participants took part in this study after giving informed consent. When giving informed consent, the participants were informed about important points including: participation was voluntary; anonymity of the participants and the confidentiality of the information were guaranteed; the right to withdraw from the research at any time; the interviews would be recorded, if they permitted. Also, the probable duration of the interview was communicated in advance to the participant and the interviews were digitally recorded with the informed consent of the participants

**Trustworthiness**

In this study, to validate the results, and the rigor of research, the Lincoln and Guba metrics were used. The methods were applied in the study and included: continuous observation, allocation of sufficient time for 12 months to collect data and good communication with the participants, as well as spending a lot of time to understand the concepts derived from the study, having a working background as a researcher (MRSH), as the principal investigator, and also (DKZ) in the national Disasters and Emergencies Management Center (DEMC) for several years and using this experience to select participants. 

Also, by using observers, two of the co-authors who were experts in qualitative study (DKZ, KhN), the coding process was monitored. Also, the entire research process, including collecting and analyzing data, selecting participants, implementing and selecting the core variables, was monitored by both of them and qualified individuals in qualitative research.

**Findings**

In this study, 25 participants were interviewed: a summary of their characteristics is provided in Table 1.

**Table 1:** Characteristics of participants in the study and their organizational units on factors influencing the safety against residential building fire related injuries.

Participants (n=25)		No	percent
**Gender**	Male	22	88
	Female	3	12
**Role**	Firefighter	10	40
	Experts	4	16
	Physician	1	4
	Nurse	3	12
	Fires victims	6	24
	Witnesses of fires	1	4
**Organizational**	Ministry of Health	2	8
	Ministry of Interior	1	4
	University of Medical Sciences	1	4
	Hospital	10	40
	Fire station	10	40
	Other	1	4

"Lack of a comprehensive prevention approach to fire-related injuries" was known to be the core variable affecting the safety of residents against fires in buildings in Iran. As Table 2 shows, the factors related to fire-related injuries from residential buildings are classified in four categories and 13 sub- categories: Challenges related to the structure of the building based on three sub-categories: (Challenges related to structural factors, Challenges associated with nonstructural factors and energy resource challenges in the building); socioeconomic challenges with four sub-categories (challenges of safety culture, legislative and supervision issues, economic challenges, and low education and safety knowledge); challenges linked to building residents subjected to three sub-categories: (individual characteristics, behavioral barriers and weaknesses in risk perception); and challenges of the relief and rescue service consisting of three sub-categories: (service pattern, obstructive urban structure, and factors relating to members of the public).

**Table 2:** Factors influencing the safety against residential building fire-related injuries based on participants’ experiences in fire-related building injury prevention.

Categories	Subcategory	Code			
Building structure	Challenges related to structural factors	inappropriate interior design	low-quality construction materials	Non-standard building density	An elevator and a staircase in one place
	Challenges associated with nonstructural	Lack of installation of safety equipment (fire alarm systems and fire extinguishing systems)	Inadequate layout of the interior space	Inadequate maintenance of indoor equipment	Failure to build fire escapes
	Energy resource challenges	Capsules and gas pipelines	Unsafe electrical wiring	Unsafe electrical equipment	Factors related to other energy sources
Social factors	Challenges of safety culture	Lacks of priority for safety	Unsafe lifestyle	Poor belief in safety	The worthlessness of human life
	Legislation and supervision weakness	Few laws in relation to fires safety	Lack of effective laws	Poor law enforcement	Lack of adequate supervision in relation to fire safety
	Economic challenge	Less fires insurance	Non-payment of safety subsidies	Inability to buy safety equipment	High cost of safe energy sources
	Poor education and safety knowledge	Insufficient knowledge in rescue team	Poor education in residents of residential buildings	Insufficient knowledge and information in people	
Individual illness Individual disabilities Low age and aging Individual characteristics Residents characteristics and behavior	Individual characteristics	Low age and aging	Individual disabilities	Individual illness	
	Behavioral barriers	Individual unsafe lifestyle		Individual risky behavior	
	Low perception of risk	Poor individual belief in safety		Poor individual risk perception	
Rescue and relief services	The challenge for rescue and relief services	Weak pattern of services	Weakness of equipment and function for rescue team workers	Operational weakness of the rescuers	Weakness in the scene management
	Obstructive urban infrastructure	Heavy urban traffic	Difficult access to fire scene	No emergency lane	Inappropriate design of residential buildings
	Members of the public at the fire scene	People with poor knowledge of fire extinguishing and first aid	Intervention of people at the scene	People gathered at the scene	Poor notification to pre hospital emergency

***Challenges related to building structure***

***Challenge related to structural factors***

Based on participants` experiences, inappropriate interior design such as the failure to provide fire escapes and emergency exits, as well as the use of non-safe and low-quality construction materials, are obstacles associated with structural factors that are the most important factors in the structure of residential buildings in the fire events and their related injuries. According to participants’ experiences, little attention has been paid to the safety of building construction and fire protection standards. Accordingly, some building materials used will, in the event of a fire, allow it to spread rapidly.

..."We have an example inside a residential complex, including a 7-floor building, about 3 years ago, and one of the drawbacks that caused the spread of fire was that the elevator and the staircase were in the same place. There were no fire escapes, no smoke-proof and fireproof doors ... "(P^1^8).

"... We often see that in the construction of the roof of the newly built houses, Styrofoam is used which rapidly spreads the fires and is accompanied by a lot of hazardous smoke which is harmful for residents ... "(P 6).

***Challenges related to non-structural factors***

According to the participants, the poor design and failure to install safety equipment (fire alarm systems and fire extinguishing systems), inadequate layout of the interior space of the building and inadequate maintenance of indoor equipment are components of non-structural factors that will reduce safety for the occupants of the building. In Iranian culture, people pay a lot of attention to decorating their home, and often safety is of secondary importance. The positioning of flammable fixtures and fittings inside residential buildings near heat-generating devices such as non-conductive heaters will spread fires and increase the injuries they cause. Also, a late start to fire extinguishing as a result of being poorly informed about the start of the fire due to a lack of smoke detectors can cause fires to spread and increase the likelihood of casualties.

..."Other cases that I regrettably saw were the inadequate use of smoke detectors and extinguishing systems, and even those who are forced to use the final permission, escape the responsibilities with the use of non-standard and inexpensive equipment just to get it... "(P15).

"…Sometimes in the room, the equipment is arranged under the bulb. A few days ago we were going to visit the building, goods in the warehouse of the building were arranged close to the lamp ..." (P4).

***Challenges related to energy resources in building***

According to the participants in the study, there are considerable challenges associated with energy resources used in buildings, especially canisters and gas pipelines, unsafe electrical wiring as well as unsafe electrical equipment, were among the most important inducing elements of the fires. The point that some participants emphasized was the inappropriate storage of flammable liquids at home, especially in the periphery of the city, which increased the risk of fires and its injuries. Plugging in too many devices to one power outlet and overloading the system is one of the things that can start the fires.

..." A person rents the second floor and uses a two-branch hose for a stove, and one for samovars, while half a meter away, there is a wall heater. When he leaves the house, the gas released from the hose pipe into the water heater (flammable)... "(P 2).

" … We had some branches taken from a power outlet that caused a fire because the outlet did not have the ability to carry such a heavy load. Another discussion is storage of hazardous materials at home, for example, a taxi driver, who wants to spend less time in the queue at the gas station, will store two gallons of gasoline in the parking lot ... "(P1).

***Socio-economic challenges***

***Challenge of safety culture among the community***

This category refers to factors which can act as safety barriers against building fires. They are rooted in the cultural and social lives of the residents. Poor attention to safety and lack of a proactive attitude and belief in its value, as well as a failure to prioritize safety in people’s lives, along with their dangerous lifestyle, are some of the issues which were considered by participants in the study as an important challenge to preventing fires in housing blocks. A fatalistic attitude on the one hand and poor belief in safety was almost all mentioned by participants in the study.

..." Because most of us think that these events are for others and then we do not have that. Unfortunately, when we believe it, we have experienced it and the injured are ready to spend many dollars on burning scars, while they are not willing to spend much less to prevent it ... "(P15).

According to the participants’ experiences, the behaviors associated with fire risk, non-compliance with the safety and internal structure of the building in Iran are such that the possibility of fires is greater. For example, the use of flammable materials in Iran, such as the use of curtain and carpet decoration, can increase the possibility of fires.

..." In one case, they used gasoline for cleaning and washing inside the building and the door was closed and gas was produced and so an explosion took place ..." (P 4).

"... I went to a conference that had a lot of decorations, and it was clear that it had cost a lot to do this, but in the case of fires, safety was not observed, for example, all doors were open to the inside ... "(P6)

***Legislation and supervision weakness***

Most participants raised the issue of lack of both laws and adequate supervision in relation to fire safety activities in the building. They demanded that building safety certificates be completed before people moved into their homes. This is due to the fact that policy makers have not been adequately concerned with the drafting of a law whose purpose is to protect and enhance the safety of buildings. In many cases, accelerating the unit construction is not prioritizing safety. The lack of a law for the non-use of flammable Styrofoam or a law covering the dangers of the extensive use of domestic gas systems that increases the possibility of explosion is one of the important points that were referred to by the participants.

..." In our country, municipalities require tall buildings to have fire extinguishing systems, but not so high buildings, villas and village houses lack this requirement... "(P 12).

 "... The requirement to install safety equipment is valid only until the end of the building work, and thereafter there is no supervision ..." (P 1).

On the other hand, in the current situation in Iran, the restoration of buildings and the mass construction have become common in recent years. In construction, less engineering supervision in the construction of buildings and inadequate supervision or lack of presence in the building, less qualification in building construction and the lack of strong laws that prevent these problems are important points mentioned by participants in the study.

***Economic challenge***

Issues and topics related to financial problems were raised as socioeconomic challenges. Participants mentioned lack of fire insurance, non-payment of safety subsidies, inability to pay, the high cost of some energy sources compared to others such as electricity compared to gas that contribute to the safety of residents. Participants say fire insurance of the building prevents residents from suffering great financial losses in the event of fires, which means the residents immediately leave the fire scene fewer worries about their property.

..." Sometimes, instead of using 2.5mm electrical wire and because of the cheapness of 1.5mm electrical wire, they are used, and since they are weak, they will soon come into contact with the fires ..." (P7)...." If a home is insured against a fire, we are not worried about the property damage caused, because many people who extinguish the fires are worried about the economic losses ..." (P 12).

***Weakness of education, knowledge and attitudes to fire safety***

Many participants pointed out that there is a less education, and insufficient knowledge and information in residents of residential buildings that could have a negative effect on their behavior during the fires and cause increased injuries. Also, firefighters lack medical knowledge to treat injuries. For this reason, the probability of there being casualties increases if there is an increase to the relief and rescue time. In schools, fires prevention training is given low priority. On the other hand, the lack of this training, coupled with the inadequate attitude to the need for safety, is an issue that affects safety-related activities and efforts.

..." We had a fire but the extinguisher which was in front of their home, was not used because of lack of training. Therefore, training is important ... "(Participant 5).

"... When a housewife uses an electricity outlet, she does not know how long to use it. She uses it 20 minutes continuously and then it becomes overheated... "(P 14).

Firefighters who are able to enter the fire zone do not have sufficient knowledge to revive and treat injured patients because they lack medical knowledge ... "(P1).

***Challenges related to residents characteristics and behavior***

***Individual characteristics***

Individual characteristics such as low age, aging, illness and disability are barriers to the safety of fire-related injuries that most participants mention. Buildings are not designed to meet the needs of people with disabilities and handicaps. Among the individual characteristics, the lower age, especially under 5 years, aging, especially over 70 years, and individual disabilities and deficits such as deafness, blindness and psychotic problems are the most important safety barriers attributable to individual characteristics that can increase injuries by fires. In fact, based on the experiences of stakeholders, the structure of existing buildings is not intended to protect the safety of ordinary people, especially those with special needs, such as children and the elderly.

"... About one month ago, an old man and woman were inside the residential unit and midnight, living room and back room underwent fires. The man and woman did by not notice the fires, due to poor hearing and loss of smell ..."(P1).

"... A fire caused by fireworks used by two kids aged 3 to 6 that had started in the bedroom and eventually spread ..."(P 2).

***Behavioral barriers***

Human behavior during fires is the most important aspect of safety for the residents of a building. The motivation to start evacuation on discovery of fires and how to respond to fire cues play an important role in their safety. As participants pointed to frequently, the risky behavior of residents living in residential buildings due to their lack of interest in safety, is one of the most important risk factors for their health and one of the barriers to prevent injuries caused by fires. 

"…Due to the unhealthy behavior of an individual, he starts to run away after a fires and suffers from 70% burning. Despite the fact that his friend recommends that he lies down and rolls, he does not pay attention, which makes him unfortunately pass away after admission to hospital ... "(P 15).

"... Other things that are strange in 2017 is the use of gas cylinders to open the sewer pipe by a number of people, which causes gas leakage in several floors and a large fires ..." (P 15).

***Low perception of risk***

Participants in this study highlighted the unawareness of the risk of fires among residents living in residential buildings along with poor understanding of the dangers and injuries caused by the fires, as well as their belief that such incidents would not happen. They considered low risk perceptions to increase the likelihood of fires and their related injuries. People seem to have a low understanding of the dangers, and they often perform risky behaviors with a fatalistic approach.

... " there was a mother putting her child to sleep next to the heater after the baby had had a shower, leaving one side of the blanket on the heater, and the other side thrown over the baby to get her baby warm and then went out of the room ..." (P9. (

"… for example, the winter is over and the stove was removed, but the cap is not placed on the valve pipe, and the handle is on it, and we had a case when the resident knocked the gas valve when the equipment was moved, and the valve opened a bit because it had no lid. Gas leaked and it exploded with the first spark ..."(P 5).

***The challenge for rescue and relief services***

This category includes the following sub-categories of barriers preventing the relief services from reaching the building to extinguish the fire and rescue the injured. They include: poor service patterns, obstructive urban structures, and factors related to members of the public. After the fires, any challenge in the provision of services can be associated with the severity of the fires, and in particular the injury and increased mortality resulting from it.

***Weak pattern of services***

The service pattern was one of the challenges identified in the aftermath related to the rescue and relief services that participants pointed out during the fires in the buildings. Based on participants' experiences, the inconsistency in the management of the scene, issues on the technology of relief agencies, both in the field of firefighting equipment and in pre-hospital care, have been some of the major challenges facing pre-hospital services. The scientific and operational weaknesses of the rescuers are one of the explanations for this category.

***Weaknesses in the scene management***

Sometimes there is interference in the firefighting task by members of the public along with the poor coordination between the agencies providing relief services and also serving simultaneously as firefighters and personnel of pre-hospital emergency. In some cases the cooperation between police and rescue agencies causes special problems at the fire scene. There is no clear command structure as to who is in charge of coordinating the rescue efforts. This problem can also aggravate the injury rate and waste both time and money.

..." We, as the emergency staff, are at the front of the building on fire to help victims, but the firefighters take the victims out from the rear door... "(P 1).

***Deficiencies of equipment and function for rescue team workers***

Problems associated with the lack of necessary and adequate equipment for the rescue operation by the firefighters and the pre/hospital emergency care are categories that were based on the experience of the participants. The sub-standard performance of rescuers in different organizations impacts on safety. For example, the golden time for rescuing those trapped on fire and smoke is very short and in this limited time, medical personnel are not able to enter the scene and firefighters lack the ability to deal with the fire victims, which can increase both injury and death.

"…Emergency staff (such as firefighters) lack protective equipment such as clothing and oxygen to enter the hot zones ..." (P1). "…Our firefighters who are able to enter the hot zone are not adequately trained for resuscitation and treatment of fire victims..." (P1).

***Poor urban infrastructure***

Participants in this study commented that the urban traffic, difficult access to adjoining streets, inappropriate design of residential buildings, inappropriate rescue and relief bases as well as lack of an emergency lane in the road system are the other challenges for rescue services when fighting a fire. The obstacles associated with the urban infrastructure lead to an increased response time and create a disruption in providing an optimal firefighting and emergency medical service. Large residential complexes in case of fires need large vehicles and fire lifts to be able to extinguish the blaze. Unfortunately, some complexes and high-rise buildings have been built in areas where the narrow streets makes it difficult to provide help. In addition, cars parked in the route of the emergency services lead to a major problem for passing vehicles, especially during the night and this situation makes things far more difficult for the firefighters. This delays the firefighting and rescue operation which probably will increase losses. The weakness in the public transportation infrastructure in the study setting forces people to use private vehicles, bringing about heavy traffic in the streets which means that rescue vehicles do not reach the scene on time.

..." In a street planned for 10 families, 200 and even more families have settled. Especially if fires happen at night and the cars are all parked on the streets and their owners are not present ... " (P 1).

"…One of the barriers to providing help is the dense urban residential buildings. For example, in an 8-meter alley ten-story apartment blocks have been built which, in the case of fires, makes it difficult for vehicles to provide help for the upper floors…" (P 1).

"… The problem of urban traffic is the inadequate development and modernization of public transport; the metro and buses are totally inadequate and it is difficult for passing emergency vehicles when needed..." (P 1).

***Intervention of people at the scene***

According to many of our interviewees, the gathering and involvement of members of the public who lack knowledge of fire extinguishing and first aid at the time of the fires in a building can lead to more problems. It can make relief work more difficult and the timing and quality of these services will be disrupted. The problem of people gathering at the scene to provide help or simply out of curiosity is not only seen in the fires but also in other emergencies and always leads to extended response time. So, it seems, the police need to create an action plan for all emergency events, according to which the presence of people or individuals unrelated to the disaster relief is prohibited.

..." Numerous crowds gather at the site and we have no coherent plan for the coordination of these groups ..." (P 1).

 "…The error that occurs is when we hear fire warning system, we try to extinguish the fires ourselves whereas the firefighting services must be called in.... "(P12).

1. Participant

## Discussion

According to the research team, this paper, as the first qualitative study on the safety obstacles regarding fire-related injuries in residential building in Iran, was carried out using grounded theory method. This study aims to explain the factors which impact on the prevention of fire-related injuries in the context of fires in residential buildings. It is based on the experiences of stakeholders who explain the major barriers to preventive activities in this context. The core variable which covers all sub-categories in the study was the lack of a comprehensive approach to the prevention of fire-related injury. This core variable covered the main categories, namely "challenges associated with the structure of the building; socio-economic challenges; challenges related with residents of the building and challenges of rescue services".

"Lack of a comprehensive approach to the prevention of fire related injury" was the most important barrier in this study. Lack of such an approach leads to a failure to take into account human vulnerability during fire incidents. Accordingly, when designing residential areas adequate access for emergency services in the case of a fire should be taken into account in order to decrease the severity of injury or death. One of the important factors that affects this approach is a respect for the value of human life, which makes the system designer prevent the incident or reduce its severity. Countries with a planning process for the suitable deployment of urban systems are more successful in preventing the deaths from fire events.

Challenges associated with building structure, such as inappropriate interior design and use of low-quality construction materials are problems that increase fire-related injuries. The reason for this is based on various data extracted, the most important of which can be the inadequate supervision of engineers, economic problems and a low prioritization of safety in the community. Designing safe buildings by measures such as including fire escapes and the use of high-quality fire-resistant materials can prevent injury caused by the fires. Lack of a comprehensive approach to prevention of fire-related injuries is the major issue that is followed by an inappropriate and unsafe design. It seems the greater the number and size of the exits, the lower the time for the occupants to leave the building safely. According to James Patterson’s book, "Simplified Design for Building Fire Safety" from Canada, the resistance of building materials against fires is of particular importance to prevent the outbreak and spread of fires.^22^

Another challenge related to the building that was mentioned by most participants is the inadequate installation of smoke and heat detectors as well as fire extinguishing systems. One of the most important components in safety promotion and fire-related injury prevention is the notification of the emergency service about fires at the earliest opportunity. This helps residents to escape quickly and thus saves lives and reduces the injuries. Most studies have also pointed out the role of these systems in promoting the safety of residents.^1,23,24^ It seems that plans to install more of these systems in LMICs countries should be considered seriously.

Inadequate layout and maintenance of interior equipment and devices were also fire risk factors. This causes the fires to spread rapidly, causing more injuries by smoke and heat. To prevent it, non-flammable equipment and safety equipment should be used and placed at a safe distance from the heat supply. A study that was conducted at Lund University, attributed the rapid spread of fire in residential buildings to abundant flammable materials such as furniture, bedsteads, clothing, and interior wall coverings.^25^ The low-perceived risk of fires is one of the important factors and is followed by the inappropriate installation and maintenance of equipment. Other factors associated with the building are those associated with energy resources, such as gas, electricity, and flammable liquids at home, as mentioned by most of the participants.

The use of gas capsules, burners, gas and oil heaters has become outdated in many countries over the past decades, but unfortunately in LMICs countries including Iran, it is still in use which causes an increase in casualties. The World Health Report: 2004 also emphasized the use of heating and oil burners in the onset and spread of fires in LMICs.^26^ Therefore, instead of using hazardous energies, using electric thermal equipment and minimizing the number of energy resources in residential buildings can be safer.

Furthermore, flammable liquids such as gasoline and petrol must be kept away from the home, so that in the event of a fire flames do not enter the interior home space. Keeping these items in homes as a source of fires has also been emphasized in other studies in Iran, which caused fire-related injuries. The study conducted by Rezapur-Shahkolai et al, in Tuyserkan City, Iran also pointed to the role of fires and injuries by gas capsules especially in rural areas.^27^ The other study, conducted in the north of Iran, also commented on the role of gas and oil supplies as well as the use of oil-fired devices.^28,29^

Socioeconomic challenges such as low safety culture, deficiency of legislative and supervision, economic problems, and inadequate training and safety knowledge were mentioned by most participants. A study conducted in Canada showed that mortality rates are far higher among people with lower socioeconomic status, ^30^ which could be due to low knowledge and financial inability to provide safe equipment. The unsafe lifestyle, the lack of prioritization and low belief in safety in the community, as the challenges of safety culture, were reported as a major barrier by most participants in this study. The results of the research also showed that knowledge, attitude and belief have a positive relationship with safe behavior and lifestyle against fires.^31^ To boost these factors, major attention should be paid to increasing the risk perception among residential building occupants. The lack of both laws and clarity as to the main responsibility for monitoring residential buildings are other factors which can lead to such fires. Countries that have better strategies and practices in fires prevention programs have adopted laws to enforce the installation of automatic fire detection at national and local levels. These laws and supervision have led to a reduction in casualties caused by fires in buildings.^32^ However, a better understanding of risky behaviors which should be avoided is an important factor for fire-related incidents and injuries in Iran.

The economic problem is one risk factor for fires and the safety barriers for occupants of residential buildings considered by many participants. One study also asserts that fires have greater impact on low-income areas.^33^ Since the construction, equipment and the use of safety devices, as well as building fire insurance are expensive, many low-income areas cannot afford them. Therefore, governments must identify these groups and pay subsidies to make them insured against fires. A study conducted in United States suggested distributing smoke and heat detectors to prevent fires and promote residents’ safety.^34^ Obviously, in all protection issues insurance is a pivot point because many of the people who are at the incident scene and try to extinguish the fire are worried about the economic damages.^35^ The problem they are facing can be resolved by fire insurance for their homes and this can prevent people from being harmed.

The low level of training for relief and rescue teams and the general public, as well as poor knowledge and safety information were mentioned by many participants. It seems that the knowledge and expertise of the relief and rescue services are not very satisfactory. Also, the public’s safety awareness is low about building fires. According to Fredric's study, two thirds of the population are not aware of safe behavior during fires.^36^ It implies a great need for more activities for safety awareness in a society like Iran. Many of the fire-related injuries and casualties can be reduced by training and increasing the awareness of how to escape and exit from the place of fires.^37^ Therefore, public education and raising awareness of society in various ways are one of the most important preventive activities for the residents' safety of buildings against fires. One study in Germany also found that in many cases educating people in emergency first aid is helpful.^38^

Individual characteristics such as age, illness, and disabilities, as well as behavioral barriers and low risk perceptions were among the challenges faced by residents of the building as mentioned by many interviewees. It seems that some age groups such as children and the elderly are at increased risk of fires due to cognitive and physical inability. Also, some fire victims and people with disabilities, such as the deaf and those with mental illness, are not able to understand the warning from fire alarm systems. Many studies have shown that in the event of a fire, mortality rates are higher in children and the elderly.^30,36,39^ It is also emphasized that in order to increase safety, elderly people living alone should be identified and prioritized. Moreover, installing smoke detectors, an emergency exit plan for families, especially the elderly and children, should be created.^40^ Also, the risky behavior of residents during the fires as well as unsafe lifestyle and poor confidence in safety are mentioned as safety barriers by almost all participants. Risky behavior can be due to poor safety knowledge, but attitudes and beliefs of individuals largely shape how they behave. Therefore, promotion of safe operation requires a national determination and will to provide continuous and effective training, special programs to change attitudes and eventually residents' performance towards safe behavior. Miller's study conducted in New Zealand also emphasizes that the most important cause of fires is human errors or risky behaviors.^10^ This also argues for the importance of safety awareness in society and activities toward their positive attitude for safety behavior.

Several studies have identified risky behaviors as one of the barriers of individual safety.^12,41-43^ A study conducted in United States shows that in order to reduce fire-related injuries, there must be planning to control and reduce risky behaviors.^42^ Risk perception can affect the individual behavior and show how individuals decide to eliminate hazards. Manuela's study emphasizes that people's risk perception is affected by various factors such as psychological, social, physical, normative issues, and the cultural factors of the communities in which they live.^44^

The prolongation of the time needed for extinguishing fires and relief efforts led to increased mortality rates as reported by some of the participants in this study. The weaknesses and inefficiencies of relief and rescue workers, inadequate relief supplies and weaknesses in the coordination and management of the incident scene as well as obstructive urban structures were the main reasons for this problem. It seems that the upgrading of technology and equipment for the firefighting services and other relief forces can play a role in reducing fires, so that the process as the fall in the number of fires in residential areas of high-income countries can be attributed to many factors, including the improvement of methods and technologies in fires and rescue services.^45^ Other causes for the prolongation of the relief and rescue period include the inadequate urban structure, such as difficult access to the adjoining streets. As mentioned in Mobley`s study, in buildings with high population density due to the difficulty of emergency evacuation, can lead to an increase in injury and mortality.^42^

Urban traffic has also been referred to as a relief barrier by participants. Two other studies in Iran also mentioned urban traffic as one of the obstacles to relief operations. ^46,48^ The gathering and involvement of members of the public at the fire scenes is one of the barriers to the firefighting and emergency pre-hospital medical care. Volunteers in these cases can be better managed by relief forces and police and their ability and capacity can then be used to assist and reduce casualties. Unfortunately, due to the less comprehensive management approach at the incident scene, this opportunity becomes a challenge that prevents relief operations. The problem of obstructive urban structure and the problems of access to relief operations and rescue organizations are topics that have been mentioned in other studies in Iran.^35,47^ The problems of the city's inadequate structure on one hand and the high traffic congestion, especially in the metropolitan areas of Iran on the other hand, are factors that aggravate the injury not only in the fires, but also in other types of incidents that policymakers need to pay attention to. Again, all kinds of injury prevention needs a systematic approach for better management and prevention, which in other kind of study were also under emphases.^49^

**Limitations and strengths of the study**

The study, which was based on interviews gathered various stakeholders’ experiences including firefighters, medical staff, fire victims and those who had experienced fires in relation to the factors affecting the safety of building residents against fire. All participants were well experienced and knowledgeable and saturation was reached for all the concepts. Fresh perspectives emerged due to these techniques and using constant comparative analysis, a result which indicates the strength of the study. Due to the impossibility of selecting participants in the overall geographic scope of the research, the generalization and application of the results should be more precise. However, the authors have never sought generalizations. Sometimes coordination was needed to adjust the interview time with some officials, which led to a prolonged process of collecting data. Interviews with some hospitalized fire victims were difficult due to their physical condition. Both of these cases were resolved by rescheduling the time.

## Conclusion

Lack of a comprehensive approach to the prevention of fire-related injuries is the most important barrier to fire-related injury prevention that affects most factors including challenges related to building structures, socioeconomic factors, human factors as well as challenges of merging the efforts of the rescue services which eventually should be considered. Therefore, safety promotion requires a comprehensive and preventive approach and also reforms in various sectors, such as: building, legislation, culture and individual behaviors. 

**Acknowledgment**

The author would like to thanks firefighters, physician and nurses involved in the provision of the data in this study.
